# Editorial: Exploring immune-stromal cell dynamics: pathways and therapeutic implications

**DOI:** 10.3389/fimmu.2025.1744273

**Published:** 2025-12-15

**Authors:** Stacy Ryu, Stephen B Gauld, Matthew M Staron, Ian D Odell, Jenna L Cash, Timothy RDJ Radstake

**Affiliations:** 1 Abbvie Inc., North Chicago, IL and Cambridge, MA, United States; 2 Department of Dermatology, Yale School of Medicine, Department of Immunobiology, Yale University School of Medicine, New Haven, CT, United States; 3 Centre for Inflammation Research, Institute for Regeneration and Repair, University of Edinburgh, Edinburgh, United Kingdom

**Keywords:** tissue resident immune cells, fibroblast and immune crosstalk, tertiary lymphoid structures, cell-cell interactions, mesenchymal stem/stromal cells

Bidirectional immune-stromal cell dynamics play a pivotal role in immune homeostasis and disease pathogenesis. Accumulating data demonstrates that stromal cells are more than mere structural components; rather, they constitute a key part of the immune system. Specifically, stromal cells have been shown to shape tissue homeostasis, inflammatory responses, and disease progression in various tissues - including pulmonary, lymphoid, and vascular systems. Advances in omics technologies (single cell and spatial transcriptomics) have uncovered the molecular phenotypes of stromal cells and illuminate their potential as key immune sentinels, capable of regulating the inflammatory milieu by detecting danger signals and releasing soluble mediators. Novel bioinformatic tools that enable analyses of complex receptor-ligand interactions have further uncovered how dysregulated crosstalk between immune and stromal cells can drive the initiation and progression of such disorders. Given their role as orchestrators of the immune response, stromal cell types are investigated both as therapeutic targets and as therapeutic modalities/approaches. The current Research Topic aims to deepen our understanding of the molecular profiles, mechanisms and pathways of stromal cells that govern immune cell regulation, while shedding light on their therapeutic potential.

Yasmin et al. provided a comprehensive review on the architectural and functional diversity of stromal subsets in secondary lymphoid organs. The authors summarize key findings on the heterogeneous molecular phenotypes of distinct stromal populations such as fibroblastic reticular cells (FRCs), follicular dendritic cells (FDCs), marginal reticular cells, and adventitial cells in lymph nodes, spleen, and Peyer’s patches. These subsets occupy discrete anatomical niches throughout secondary lymphoid organs and provide scaffolding critical for immune cell migration, antigen presentation, germinal center (GC) formation and orchestration of adaptive responses. Notably, fibroblasts are emerging as immune sentinels that express pattern-recognition receptors, shape cytokine and chemokine milieus, and regulate central and peripheral tolerance. For example, fibroblast subsets and FDCs modulate germinal center formation, supporting various aspects of B cell development and antibody responses. During infection or inflammation, fibroblast subsets in SLOs respond by remodeling the extracellular matrix (ECM), upregulating inflammatory mediators and modulating the activation threshold of T cells.

Advancements in omics platforms and integrative data analyses of disease samples and preclinical models continue to elucidate the role of immune-stromal interactions in shaping pathogenic microenvironment during disease. To this end, Liu et al. utilized single-cell RNA sequencing to provide an in-depth atlas of cell populations within abdominal aortic aneurysm (AAA) using a classical preclinical mouse model. The study detected fibroblast subtypes with inflammatory and distinct metabolic phenotypes unique to AAA, expressing Apoc1/Fabp4 (lipid metabolism) and various immune activating factors such as CCL, TGFβ, and TNF. Pseudotime and trajectory analyses, along with cell-cell interactome analyses suggested that fibroblasts play a crucial role in driving the Trem2- positive macrophage population, a dominant inflammatory effector cell type that may perpetuate vessel wall injury and aneurysm progression.

Under pathogenic conditions such as chronic inflammation, mesenchymal cells can be derived from epithelial origin through epithelial-mesenchymal transition (EMT). Zhang et al. summarizes the field of EMT as a key pathophysiological process underlying irreparable airway remodeling in asthma. Chronic allergic inflammation, eosinophil infiltration, and repetitive epithelial injury activate EMT via TGF-β, Wnt, Notch, and MAPK signaling cascades. This leads to the loss of epithelial integrity, enhanced generation of migratory fibroblast and myofibroblast, and accumulation of extracellular matrix, perpetuating fixed airflow obstruction. Additional mechanisms such as microRNA regulation and autophagy affect EMT, airway remolding and fibrogenesis. Although knowledge gaps remain - including exact triggers, genetic/epigenetic regulation and reversibility of EMT - this study highlights the therapeutic potential of EMT related pathways for asthma, addressing airway remodeling and fibrosis.

The unique and pleiotropic ability of stromal cells to regulate inflammatory, fibrosis, and wound repair pathways underscores their therapeutic potential in various diseases via multipronged mechanisms of action. Hazrati et al. provide a seminal review of mesenchymal stem cell (MSC)-based therapies for a spectrum of lung diseases, including COPD, asthma, idiopathic pulmonary fibrosis (IPF), and viral infections. In contrast to traditional anti-inflammatory drugs, which often fail to restore immune homeostasis or regenerate lost tissue, MSCs - through secretion of cytokines, growth factors and exosomes - can drive robust immunomodulation, tissue regeneration, and reversal of fibrosis. Preclinical studies have demonstrated the ability of MSCs to dampen pathological neutrophil and Th17 responses, enhance Treg function, and skew macrophages toward a reparative M2 phenotype. Despite remaining challenges such as ensuring targeted MSC migration, refining exosome engineering, and evaluating long-term safety in chronic diseases, the pleiotropic effects underscore the promise of MSC therapies in delivering transformative therapies for lung diseases.

Collectively, this Research Topic highlights a paradigm shift: mesenchymal and stromal cells are dynamic regulators, not mere structural bystanders, in both tissue homeostasis and immune dysfunction. Advanced molecular and single-cell analyses now enable precise mapping of cell heterogeneity and interactions, supporting the design of novel immunomodulatory and anti-fibrotic interventions. Future progress in treating chronic inflammation, fibrosis, and immune dysregulation across various diseases will increasingly depend on our ability to understand and therapeutically manipulate the mesenchymal and stromal landscape.

